# Variability in the virulence of specific *Mycobacterium tuberculosis* clinical isolates alters the capacity of human dendritic cells to signal for T cells

**DOI:** 10.1590/0074-02760190102

**Published:** 2019-08-12

**Authors:** Ana Gabriela Ramos-Martinez, Monica Alejandra Valtierra-Alvarado, Mariana Haydee Garcia-Hernandez, Rogelio Hernandez-Pando, Julio Enrique Castañeda-Delgado, Céline Cougoule, Bruno Rivas-Santiago, Olivier Neyrolles, Jose Antonio Enciso-Moreno, Geanncarlo Lugo-Villarino, Carmen Judith Serrano

**Affiliations:** 1 Instituto Mexicano del Seguro Social, Unidad de Investigación Biomédica Zacatecas, Zacatecas, México; 2 Universidad Autónoma de San Luis Potosí, Escuela de Medicina, Departamento de Inmunología, San Luis Potosí, México; 3 Instituto Nacional de Ciencias Médicas y de la Nutrición Salvador Zubirán, Departamento de Patología, Sección de Patología Experimental, Ciudad de México, México; 4 Catédras Consejo Nacional de Ciencia y Tecnología, en Instituto Mexicano del Seguro Social, Unidad de Investigación Biomédica Zacatecas, Zacatecas, México; 5 Université de Toulouse, Institut de Pharmacologie et de Biologie Structurale, Centre National de la Recherche Scientifique, Université Paul Sabatier, Toulouse, France

**Keywords:** tuberculosis, dendritic cells, virulence, apoptosis, antigen presentation

## Abstract

**BACKGROUND:**

Once in the pulmonary alveoli, *Mycobacterium tuberculosis* (Mtb) enters into contact with alveolar macrophages and dendritic cells (DCs). DCs represent the link between the innate and adaptive immune system owing to their capacity to be both a sentinel and an orchestrator of the antigen-specific immune responses against Mtb. The effect that the virulence of Mtb has on the interaction between the bacilli and human DCs has not been fully explored.

**OBJECTIVE:**

To evaluate the effect of Mtb virulence on human monocyte-derived DCs.

**METHODS:**

We exposed human monocyte-derived DCs to Mtb clinical strains (isolated from an epidemiological Mtb diversity study in Mexico) bearing different degrees of virulence and evaluated the capacity of DCs to internalise the bacilli, control intracellular growth, engage cell death pathways, express markers for activation and antigen presentation, and expand to stimulate autologous CD4^+^ T cells proliferation.

**FINDINGS:**

In the case of the hypervirulent Mtb strain (Phenotype 1, strain 9005186, lineage 3), we report that DCs internalise and neutralise intracellular growth of the bacilli, undergo low rates of apoptosis, and contribute poorly to T-cell expansion, as compared to the H37Rv reference strain. In the case of the hypovirulent Mtb strain (Phenotype 4, strain 9985449, lineage 4), although DCs internalise and preclude proliferation of the bacilli, the DCs also display a high level of apoptosis, massive levels of apoptosis that prevent them from maintaining autologous CD4^+^ T cells in a co-culture system, as compared to H37Rv.

**MAIN CONCLUSIONS:**

Our findings suggest that variability in virulence among Mtb clinical strains affects the capacity of DCs to respond to pathogenic challenge and mount an immune response against it, highlighting important parallels to studies previously done in mouse models.

Tuberculosis (TB) remains a challenging infectious disease and was rated by the World Health Organization in 2017 as one of the top ten causes of death worldwide. In 2019, TB was responsible for more deaths than HIV and malaria, accounting for 1.6 million deaths worldwide.^1^ Therefore, there is a need to better understand the interaction between *Mycobacterium tuberculosis* (Mtb) and host cells in order to design better preventive and therapeutic strategies.

TB is mainly transmitted among humans when Mtb is inhaled via the aerosol route.^2^ Once in the pulmonary alveoli, Mtb enters into contact with alveolar macrophages and dendritic cells (DCs), which are the dominant cell targets for this pathogen.^3^ In particular, DCs represent the link between the innate and adaptive immune systems due to their capacity to act as both a “sentinel” and an “orchestrator” of the antigen-specific immune responses against the bacilli. As a “sentinel”, DCs patrol peripheral sites and are capable of recognising and internalising microorganisms that invade the mucosal barrier.^4^ As an “orchestrator”, DCs efficiently acquire and process Mtb-specific antigens at the site of infection, and then traffic them to the lymph nodes, where T-cell priming occurs. There is a keen interest in the field to investigate in detail how Mtb interacts with DCs, and how this interaction can be modulated for the efficient elimination of Mtb infection.

Upon encountering and internalising a pathogen, DCs must activate an intracellular microbicidal environment to restrict pathogen growth and spread.^5^ *In vitro* studies in monocyte-derived dendritic cells (MDDCs) suggest low levels of bacterial replication within these cells.^6^ While Mtb is known to affect cell death of infected DCs,^7^ ^,^ ^8^ the pathways related to this process have not been extensively studied in DCs within the context of TB. Upon neutralising the intracellular pathogen, DCs undergo a maturation process that allows them to process antigens derived from the pathogen, and subsequently relay this information to naïve T cells in the form of antigen presentation. To counteract this process, Mtb impairs DC maturation, reduces the capacity of DCs to secrete IL-12, and inhibits the ability of DCs to stimulate T-cell proliferation.^9^ ^,^ ^10^ ^,^ ^11^ However, further studies are required to understand the degree of the effect of Mtb virulence on the capacity of the bacteria to inhibit DCs. This is precisely the focus of this study.

The relationship between the virulence of Mtb strains and their transmissibility in humans, as well as its consequences on the quality of the host immune response, has been studied, among others, by Marquina-Castillo et al.^12^. They conducted a 10-year population-based prospective study of pulmonary TB in Southern Mexico by performing an in-depth analysis of the epidemiological and clinical data of household contacts of TB patients. The authors selected a panel of isolates representing clinical and epidemiological diversity in the population of Mtb strains found in Mexico and tested them in two mouse models. Based on strain virulence, immune response (defined by cytokine expression), and transmissibility, four phenotypes were identified. The strains representing the two extremes, from hypervirulence to hypovirulence, were Phenotype 1 (strain 9005186, lineage 3, family EAI) and Phenotype 4 (strain 9985449, lineage 4, family Haarlem), respectively. On the one hand, Phenotype 1 (Ph1) was highly transmissible; it grew rapidly in the lung and caused high mortality rates in mice along with significantly more pneumonic areas, as compared to the H37Rv reference strain. In addition, Ph1 induced a poor protective immune response in the host. On the other hand, Phenotype 4 (Ph4) exhibited a long survival index, low bacillary load, and little pneumonia. Moreover, Ph4 induced a delayed acquired protective immune response.^13^ The human leukocyte response against these Mtb clinical isolate strains is still poorly understood.

Here, we focused our investigation on the interaction of Mtb clinical isolates Ph1 and Ph4 with human DCs. Altogether, our study showed that Mtb virulence has an important effect on the interaction with human DCs and the capacity of those DCs to then stimulate to T lymphocytes expansion, suggesting that these clinical isolate strains are optimal candidates for the prospective identification of Mtb genes associated with virulence and human immunogenicity.

## MATERIALS AND METHODS

*Human blood peripheral mononuclear cells, MDDCs and T lymphocytes* - Mononuclear cells were isolated from buffy coats provided by the State Blood Transfusion Center of Zacatecas, SSA (Guadalupe, Zac., Mexico) by density gradient using Ficoll-Paque PLUS (GE Healthcare, UK). The donors were healthy individuals complying with the following criteria: 18-55 years old, minimum weight of 50 kg, fasting conditions for at least 8 h, and with no history of Hepatitis type B or C, HIV/AIDS, syphilis, organ transplants, epilepsy, tuberculosis, cardiovascular disease, or cancer. Exclusion criteria included recreational drug use, mental disease, women who were pregnant or lactating, tattoos or skin perforations (12 months previous to the donation), history of diabetes, surgery, mononucleosis, toxoplasmosis or meningitis (in the last six months), having received vaccines (in the last 28 days), alcohol or narcotics use (in the last 12 h). Different sets of individuals were used for the distinct parameters evaluated in the present protocol. The number of individuals evaluated in each assay is annotated in the corresponding figure legend. Whole blood from individuals positive for tuberculin was used only for T-cell proliferation assays.

In order to generate MDDCs, mononuclear cells (5 × 10^6^) were seeded in a 75 cm^2^ flask using RPMI-1640 medium supplemented with 10% foetal calf serum (FCS) (Gibco Life Technologies, USA), 1% pyruvate (Sigma Aldrich, USA), 0.1% 2-beta-mercaptoethanol (Gibco Life Technologies, USA), recombinant IL-4 (15 ng/mL) (Peprotech Inc., USA), and granulocyte macrophage colony stimulating factor (GM-CSF, 200 ng/mL) (GRAMAL, Probiomed Lab, Mexico), and cultured at 37ºC in a humidified atmosphere at 5% CO_2_. At days 3 and 5**,** the cells were additionally supplemented with IL-4 and GM-CSF. At day 7, MDDCs were collected by centrifugation and used for functional analyses. Alternatively, mononuclear cells isolated from healthy volunteers with positive tuberculin tests (range of 15 to 25 mm) were used to generate MDDCs and to purify CD4^+^ T cells by a magnetic bead approach using the CD4^+^ negative selection kit (Miltenyi, USA) according to manufacturer’s instructions.

*Mtb strains, culture and storage* - All manipulations with the Mtb strains were performed in a dedicated BSL-3 laboratory. The Mtb strain H37Rv was used in order to establish an experimental reference for the comparative analyses done with the clinical isolate strains Ph1 (highly virulent, with no induction of protective immune response in mice) and Ph4 (less virulent, provoking a protective adaptive immune response).^12^ The strain Ph1, for spoligotyping, belongs to the family EAI and it is susceptible to all the first-line antibiotics used for TB treatment, while strain Ph4 belongs to the Haarlem family and is streptomycin-monoresistant. All strains were grown in Middlebrook 7H9 medium (BD-Diagnostic Systems, USA) supplemented with 10% Middlebrook Oleic Albumin Dextrose Catalase Growth Supplement (OADC) (BD-Diagnostic Systems, USA). Strains were grown to reach exponential phase, and culture concentrations were measured by measuring optical density at 600 nm. Bacterial aliquots were stored at -80ºC until their use. Bacillary viability was tested using the colony forming unit (CFU) assay, growing serial dilutions in 7H10 agar plates (BD-Diagnostic Systems, USA) supplemented with 10% OADC (BD-Diagnostic Systems, USA) for 14 and 21 days.

*Internalisation of Mtb and infection of MDDCs* - Bacterial aliquots (H37Rv, Ph1, and Ph4) were thawed at room temperature, and the bacterial aggregate declumping was achieved by vortexing with borosilicate beads for 5 min and centrifuging at 2040 × *g* for 5 min. MDDC infection was performed at a multiplicity of infection (MOI) of 5 (5 bacteria to 1 cell). At 2 h post-infection (hpi), MDDCs were washed with RPMI-1640 medium to remove non-internalised bacteria. At 24 hpi, MDDCs were recollected by centrifugation and fixed with 4% paraformaldehyde (PFA) for 30 min at room temperature. In order to evaluate internalisation of Mtb strains, the fixed MDDCs were centrifuged in pretreated slides (Biocare Medical, Concord CA, USA) using a Cytocentrifuge (Wescor Cytopro 7620, USA), and Ziehl-Neelsen staining was performed to identify the bacilli associated/within cells. The percentage of MDDCs with at least one associated bacterium was calculated after counting at least 100 MDDCs/sample in high power fields using a Carl Zeiss inverted Axiovert M-200 microscope (Zeiss, Germany).

*Mtb intracellular growth in human MDDCs* - The bacterial strains were cultured at 37ºC in Middlebrook 7H9 medium supplemented with 10% OADC and 0.05% Tween-80 (Sigma-Aldrich, USA). During exponential growth, the bacteria were centrifuged (2000 × *g*) for 15 min and resuspended in 1× phosphate buffered saline (PBS). Clumps were dissociated by passages through a 26-G needle, and then resuspended in RPMI-1640 medium containing 10% FBS. The mycobacterial concentration was determined by measuring optical density at 600 nm (OD600). To test the intracellular growth capacity of these strains, MDDCs were collected at day 7 of culture and seeded in 24-well plates at a density of 5 × 10^5^ cells per well. These cells were then infected with each Mtb strain individually at a multiplicity of infection (MOI) of 0.2 bacteria per cell in RPMI-1640 medium with 10% FBS for 4 h. Cells were then washed twice with 1× PBS before addition of RPMI-1640/10% FBS. At the indicated time points, the cells were lysed in a 0.1% Triton (Sigma-Aldrich, USA) lysing solution. Serial dilutions of the resulting bacterial suspension were plated on Middlebrook 7H11 solid agar supplemented with 10% OADC (BD-Diagnostic Systems, USA) and incubated for 14-21 days at 37ºC for CFU scoring.

*Cell-death assessment of MDDCs infected with Mtb strains* - To evaluate apoptosis and necrosis, we analysed MDDCs at day 7 of differentiation using the FITC Annexin V Apoptosis Detection Kit II (BD Biosciences, USA), according to the manufacturer’s instructions. Briefly, MDDCs were infected with each Mtb strain individually at a MOI of 5 in RPMI-1640 medium with 10% FBS for 4 h. MDDCs were then washed twice with 1× PBS before addition of RPMI-1640/10% FBS. After 24 h, MDDCs were washed twice with cold PBS and resuspended in 1× binding buffer, stained with 5 µL of Annexin V and 5 µL of Propidium Iodide, and gently vortexed and incubated for 15 min at room temperature, protected from light. MDDCs were then and washed using PBS and resuspended in 4% PFA for 30 min at room temperature for fixation. Finally, the viability status of all MDDC populations was acquired using the FACS Canto II cytometer (BD Biosciences, USA). For data analysis, Flow Jo VX (Tristar, USA) was used.

*T-cell proliferation assay* - The ability of Mtb*-*infected MDDCs to stimulate CD4^+^ T cells was assessed using an autologous co-culture system, as previously described.^14^ Briefly, autologous CD4^+^ T cells (the responders) were freshly isolated and purified from healthy donors positive for the tuberculin test on the day that the co-culture was initiated. A total of 1.5 × 10^4^ 5-(and 6)-Carboxyfluorescein diacetate succinimidyl ester (CFSE) stained CD4^+^ T cells (responder cells) were added to each well in 200 μL complete media on rounded-bottom, 96-well plates (BD, Pharmingen, USA). Autologous MDDCs (the stimulators) were differentiated until day 6 and pulsed for 24 h with LPS (100 ng/mL) (Sigma, Germany). Later, MDDCs were infected with each Mtb strain individually at a MOI of 0.2 in RPMI-1640 medium with 10% FBS for 4 h, washed twice with 1× PBS, harvested, and added to the responder cells at a ratio (stimulator:responder) of 1:2.5, 1:5, 1:10, or 1:20. As a control for the response to Mtb antigens, a group of non-infected MDDCs were grown until day 6 and were then pulsed for 24 h with a peptide corresponding to the partial sequence of the Mtb early secretory antigenic target (ESAT-6) protein (20 μg/mL) for 24 h. Of note, the partial ESAT-6 protein a peptide with the sequence H2N-LNNALQNLARTISEAG-COOH was synthesised at Fundación Instituto de Inmunología de Colombia, Bogotá Colombia. After six days of culture in 5% CO_2_ at 37ºC, the cells were harvested, stained, and gated for CD3 and CD4 positivity, and then analysed for dilution of the CFSE intensity by flow cytometry.

*Statistical analyses* - The corresponding statistical analysis is annotated in each figure legend. Data was compared using two-way ANOVA analysis with Bonferroni’s post-hoc test for internalisation index, Wilcoxon analysis for CFU, while comparison inside the groups was performed with the Kruskal-Wallis test and Dunn’s post-test assuming non-normally distributed data for all the other analysed variables. Statistical analysis was performed with GraphPad Prism software v.5.0 (San Diego, CA, USA). P ≤ 0.05 was considered as the level of statistical significance.

*Ethical considerations* - The present protocol was reviewed and authorised by the National Research Committee of Instituto Mexicano del Seguro Social (The Mexican Institute of Social Security, IMSS), that includes a subcommittee for ethical approval (agreement number R-2014-785-042). Informed consent was obtained from all individual participants included in the study.

## RESULTS

**Fig. 1:**
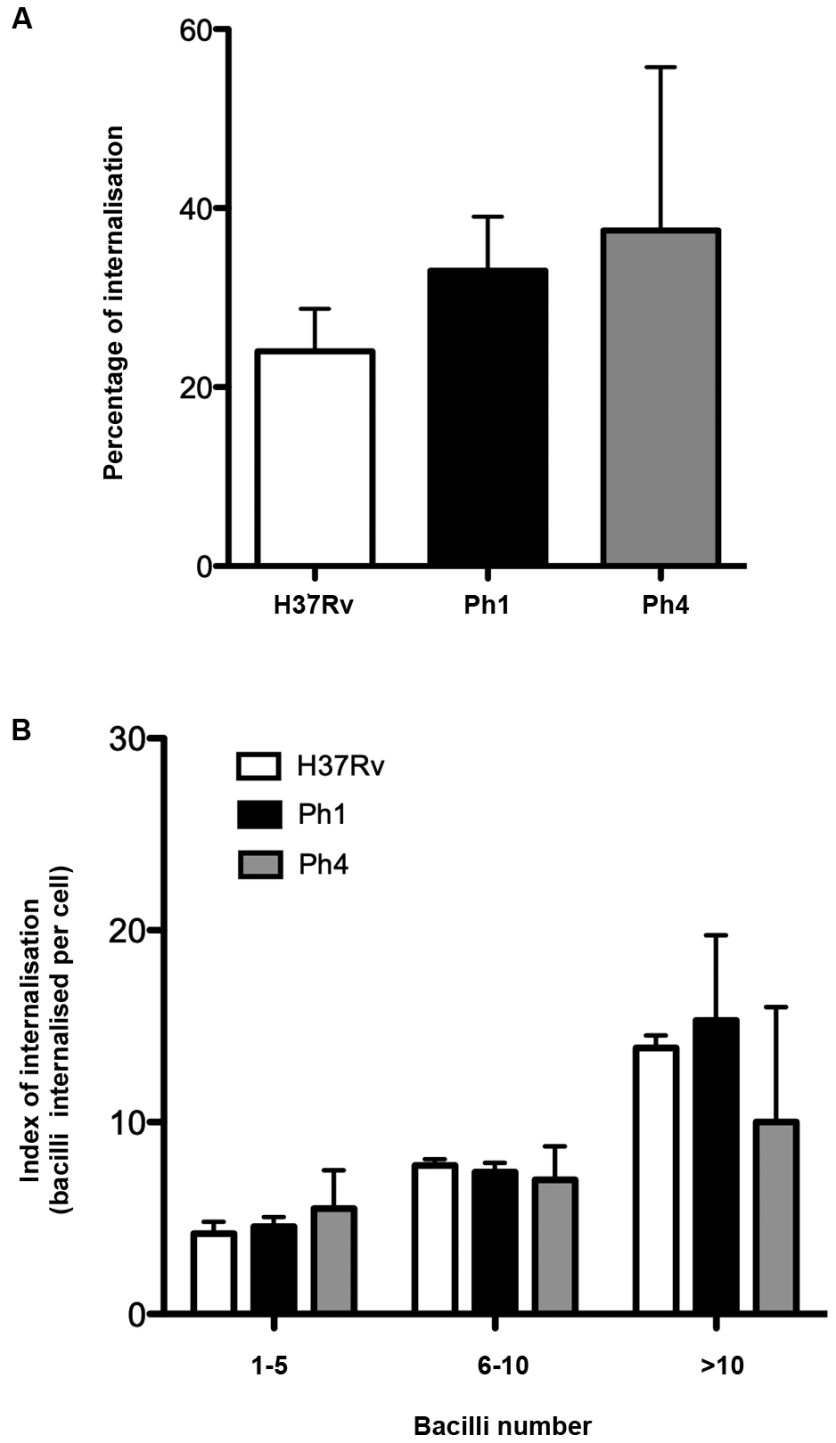
virulence of *Mycobacterium tuberculosis* (Mtb) strains does not affect recognition/internalisation by human monocyte-derived dendritic cells (MDDCs). Human monocytes were differentiated into MDDCs until day 7. (A) Percentage of Mtb bacilli internalised by MDDCs after infection with the Mtb experimental H37Rv strain (white) or clinical isolate strains Ph1 (black) and Ph4 (grey). Results were analysed using Kruskal-Wallis tests. (B) Categorisation of infected MDDCs according to the number of bacilli (1-5, white; 6-10, black; > 10, grey) internalised per cell, illustrated as percentage of infected MDDCs. Each bar represents mean ± SEM of four independent donors. Results were analysed using two-way analysis of variance (ANOVA)*.*

*Virulence of Mtb strains does not affect recognition/internalisation by human MDDCs* - In order to assess whether the virulence of the Mtb clinical isolates Ph1 and Ph4 affect their capacity to be recognised/internalised by human MDDCs, we infected MDDCs with each bacterial strain, and the bacillary load was determined by Ziehl-Neelsen staining. Our results show only a tendency towards a higher recognition/internalisation of low virulence strains compared to the reference H37Rv strain (Fig. 1A). This was confirmed by an analysis of the number of bacilli bound/internalised per MDDC, which yielded no difference among any clinical isolate compared to H37Rv (Fig. 1B). Therefore, these results suggest that variations in virulence do not significantly affect the capacity of Mtb clinical strains to be recognised or internalised by MDDCs.

**Fig. 2:**
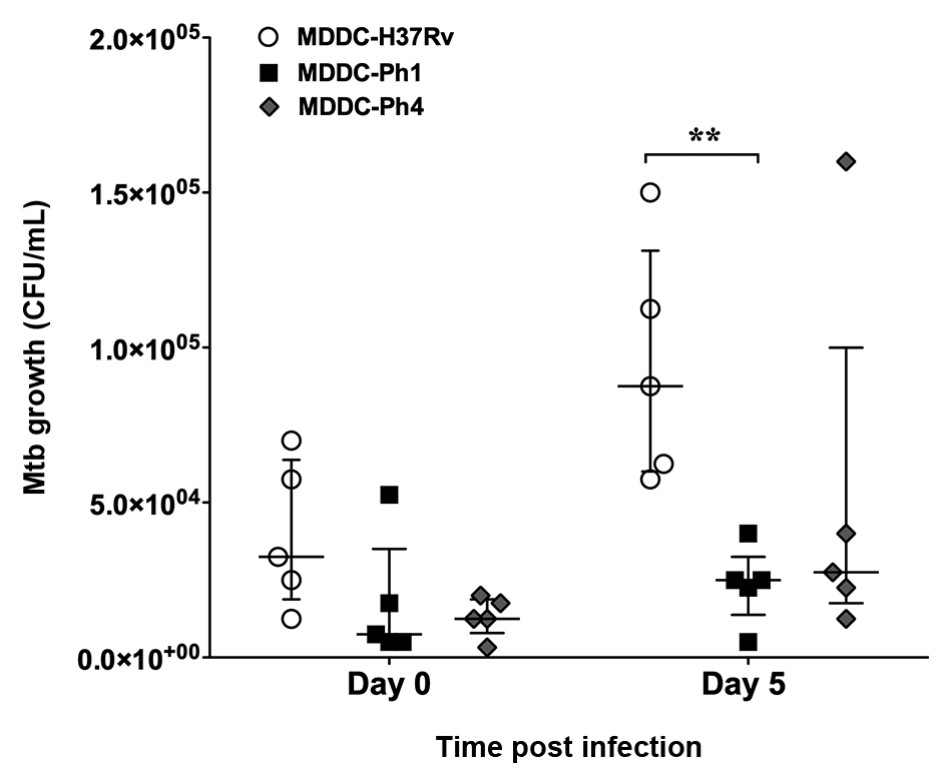
human monocyte-derived dendritic cells (MDDCs) control the intracellular growth of the *Mycobacterium tuberculosis* (Mtb) clinical strains independent of virulence. MDDCs were infected with the Mtb experimental strain H37Rv (black circles) the Ph1 clinical isolate strain (white squares), or the Ph4 clinical isolate strain (black diamonds). The intracellular growth of the bacteria was determined at 4 h (day 0) and 120 h (day 5) post-infection, as measured by colony forming unit (CFU) assays. Vertical scatter plots show the number of CFU per mL with each symbol representing a single donor run in triplicate, n = 5 different donors. Data were analysed with two-way analysis of variance (ANOVA) for repeated measures and the Bonferroni post-hoc test was applied. Bars represent median and interquartile range for five different donors. **p ˂ 0.01.

*MDDCs control the intracellular growth of the Mtb clinical strains independent of virulence* - In order to investigate whether the virulence of Mtb strains modulates their capacity to proliferate in human MDDCs, we performed CFU assays. After 4 hpi (day 0), we confirmed there was no significant difference in the bacterial charge in MDDCs infected with the different Mtb strains (Fig. 2). More specifically, we observed that MDDCs were able to control the intracellular growth of both Ph1 and Ph4 Mtb clinical strains after 120 hpi (day 5). However, at day 5, we did observe a higher inhibition of growth of the Ph1 strain compared to the reference strain (Fig. 2). Altogether, the results suggest that these Mtb clinical isolate strains fail to colonise human MDDCs, and that the difference in virulence among these strains does not play a role in this process.

**Fig. 3:**
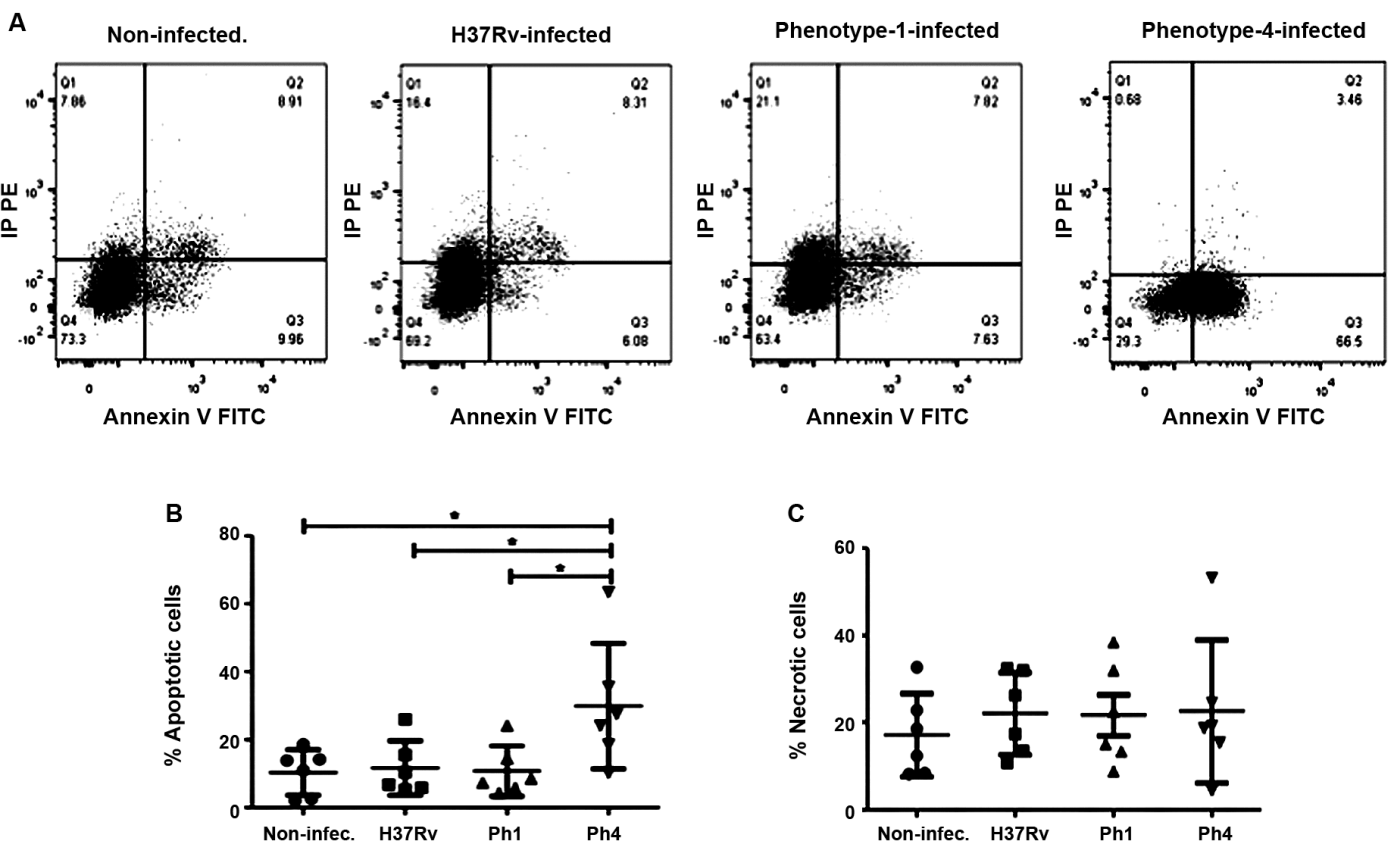
the *Mycobacterium tuberculosis* (Mtb) clinical strain bearing low virulence induces high levels of apoptosis in monocyte-derived dendritic cells (MDDCs). MDDCs were infected for 24 h with the Mtb experimental strain H37Rv, clinical isolate Ph1, clinical isolate Ph4, or uninfected control. The MDDCs were then stained using the FITC Annexin V Apoptosis Detection Kit II. (A) Representative dot plot for the gating strategy to assess MDDC cell death in non-infected, H37Rv-infected, Ph1-infected, and Ph4-infected MDDCs. The plots are illustrative of cell death measurements as analysed by flow cytometry. The amount of MDDC cell death due to (B) apoptosis (propidium iodide^-^/Annexin V^+^ cells) or (C) necrosis (propidium iodide^+^ cells) was quantified. Analysis for comparison among groups was performed in parts B and C using one-way analysis of variance (ANOVA). For the apoptosis data, Tukey´s post-hoc test was performed. Bars represent mean ± SD for six different donors. *p ˂ 0.05.

*Mtb clinical strain bearing low virulence leads to high levels of apoptosis in MDDCs* - We examined whether the difference in virulence among the Mtb clinical isolate strains affects the mode of cell death in infected MDDCs. Flow cytometry revealed that at 24 hpi, the strain with lower virulence (Ph4) induced more apoptosis in MDDCs compared to the other Mtb strains and to the uninfected cells (Fig. 3A-C). As shown in Fig. 3C, the virulence among the Mtb strains does not significantly affect the induction of necrosis in MDDCs. Overall, these results show that the hypovirulent Mtb clinical isolate strain (Ph4) differs considerably in its capacity to induce apoptosis, but not necrosis, in infected MDDCs when compared to the hypervirulent strain (Ph1) and the experimental H37Rv reference strain.

**Fig. 4:**
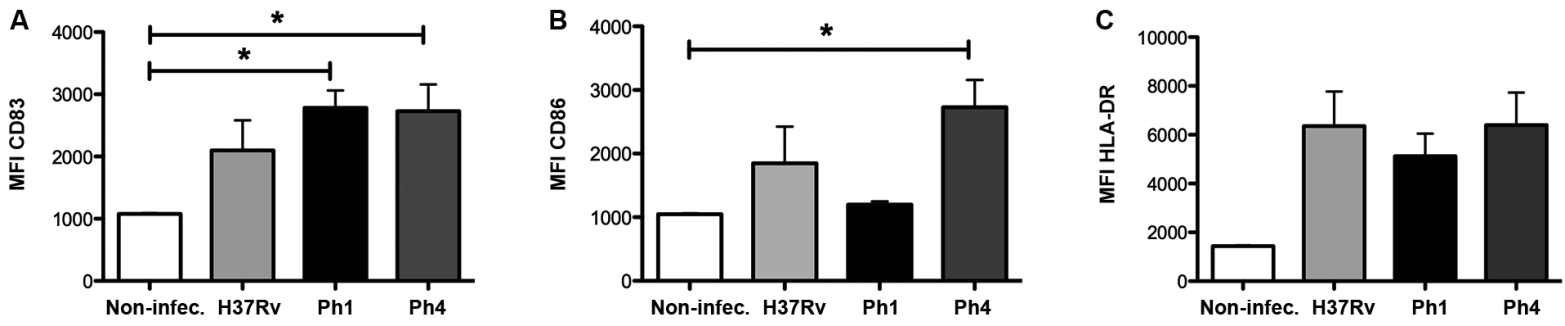
virulence of *Mycobacterium tuberculosis* (Mtb) strains does not affect the activation of monocyte-derived dendritic cells (MDDCs). MDDCs were infected with H37Rv (light grey), clinical isolate Ph1 (black), clinical isolate Ph4 (dark grey), or left uninfected as a control (white). After 24 h, MDDCs were harvested and stained for activation markers, as assessed by flow cytometry. Bar graphs show the fluorescent intensity (MFI) (mean ± SEM) for cell-surface expression of (A) CD83, (B) CD86, and (C) HLA-DR. Intragroup comparisons were performed with a Kruskal-Wallis test followed by Dunn’s multiple comparison post-test. For each group, n = 4 different donors. *p ˂ 0.05.

*Virulence of Mtb strains does not affect the activation of MDDCs* - In order to assess whether the virulence of the Mtb clinical isolate strains affects the activation of human DCs, we evaluated the expression of cell surface markers during infection by flow cytometry. As expected, MDDCs infected with any Mtb strain displayed significantly higher levels (albeit only a tendency for H37Rv) of the activation marker CD83, as compared to uninfected cells. Yet, there were no differences observed between the Mtb clinical isolate strains or compared to the reference H37Rv strain (Fig. 4A). In addition, we assessed the expression levels of receptors that are important for antigen presentation, such as CD86 and HLA-DR. Although MDDCs infected with the clinical isolate bearing low virulence (Ph4) displayed higher levels of CD86 compared to uninfected cells, we noticed there were no significant differences detected among all the infected groups (Fig. 4B). The same was true for the expression levels of HLA-DR among all the infected groups, although there was a tendency for higher expression compared to uninfected cells (Fig. 4C). Altogether, these results suggest that the difference in virulence among the Mtb strains does not affect the activation of MDDCs.

**Fig. 5:**
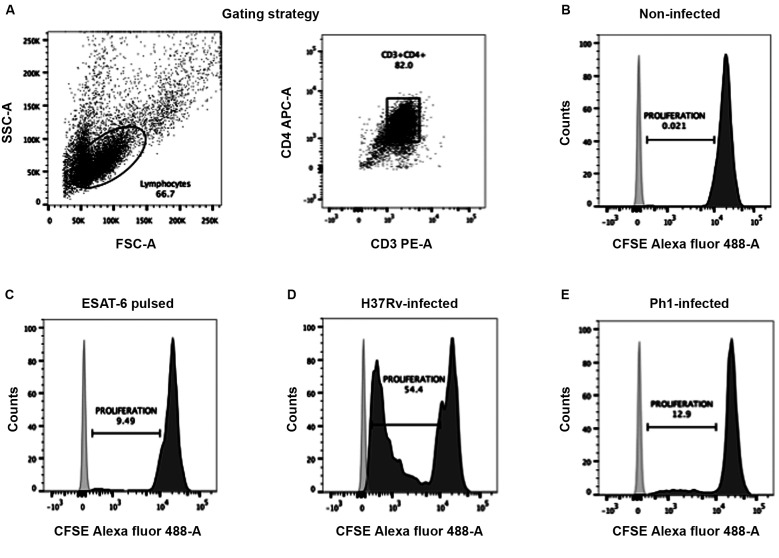
virulence of *Mycobacterium tuberculosis* (Mtb) strains affects the capacity of monocyte-derived dendritic cells (MDDCs) to activate CD4^+^ T cells. MDDCs were activated with LPS and then infected with the different Mtb strains (or left uninfected as a control). MDDCs were added at different ratios to autologous CD4^+^ T cells that were previously labelled with CFSE dye. After culturing for six days, cells were harvested and stained for flow cytometry analysis. (A) Gating strategy to select cells by size and granularity, and double positivity for the receptors CD3 (PE-A) and CD4 (APC-A) to specifically determine the T-cell population. Representative histograms showing the MFI for the CFSE dye in the T-cell populations co-cultured with (B) uninfected MDDCs (negative control), (C) MDDCs pulsed with the peptide from ESAT-6 (control for response to Mtb antigens), (D) MDDCs infected with the H37Rv strain, and (E) MDDCs infected with the Ph1 strain. The cells were plated at a ratio of 1 MDDC to 10 CD4^+^ T lymphocytes.

*Virulence of Mtb strains affects the capacity of MDDCs to activate CD4* ^*+*^ *T cells* - In order to evaluate whether the virulence of the Mtb clinical isolate strains modulates the capacity of MDDCs to activate T lymphocytes, we set up an autologous co-culture system to evaluate T-cell proliferation based on the dilution of the CFSE dye, as measured by flow cytometry. As expected, T cells that were co-cultured with uninfected MDDCs did not proliferate, as measured by the undiluted CFSE dye (Fig. 5A-B). By contrast, we observed T-cell proliferation with MDDCs pulsed with the ESAT-6 peptide, which served as positive control, at any tested ratio (MDDC:T cell), confirming the presence of an antimycobacterial immune response in healthy donors positive for the tuberculin test (Fig. 5C). Likewise, we noticed that MDDCs infected with the H37Rv reference strain were also capable of inducing robust T-cell proliferation (Fig. 5D). However, comparison of MDDCs infected with the different Mtb clinical isolates resulted in striking differences. On the one hand, MDDCs infected with the clinical isolate bearing low virulence (Ph4) were not able to prevent cell death of autologous T cells, as the co-cultures did not remain viable for the entire six day co-culture period (data not shown). On the other hand, MDDCs infected with the clinical isolate bearing high virulence (Ph1) were capable of inducing T-cell proliferation, but less efficiently than MDDCs infected with the H37Rv reference strain (Fig. 5A,D). These results were consistent for the five independent tuberculin-positive donors tested in the antigen presentation assays, even when there were not statistic differences between the rates of proliferation induced by Ph1 compared to H37Rv. Collectively, these results suggest that the virulence difference among the Mtb clinical strains is an important factor that can affect antigen presentation.

## DISCUSSION

Most of the information about Mtb strain variation and immunopathology derives from experimental animal models.^13^ Yet, this information cannot be extrapolated directly to human TB infection, and thus highlights the need to study Mtb strain variation using human leukocytes. In the present study, we investigated whether Mtb clinical isolates obtained from a prospective population-based study of pulmonary TB patients in Southern Mexico, whose virulence varied dramatically from high (Ph1) to low (Ph4) in a mouse model,^12^ differ in their interaction with human DCs in comparison to the standard experimental strain H37Rv. We believe this study makes the following contributions in advancing our understanding of how these Mtb clinical isolates differ in their interaction with human DCs.

First, we showed that the Ph1 hypervirulent strain diminishes the capacity of human DCs to activate autologous CD4^+^ T cells. Compared to DCs infected with H37Rv, Ph1-infected DCs displayed a poor capacity to induce proliferation of autologous T cells in our *in vitro* co-cultures. This is not due to a deficiency in DCs to recognise or internalize the Ph1 strain in comparison to H37Rv. Additionally, judging from the induction of antigen presenting molecules (e.g. HLA-DR, CD86) and cell death pathways (e.g. apoptosis, necrosis), we can rule out a problem with the ability of the Ph1 strain to activate DCs. Rather, we believe that the diminished capacity of Ph1-infected DCs to activate autologous T cells may be due to their inability to colonise these APCs. Based on our CFU assays, we determined that Ph1 failed to achieve a significant level of intracellular growth in human DCs. Therefore, we can infer that the Ph1-infected DCs may have less antigenic material available to present and activate naïve T cells. This is in contrast to the results obtained in the study by Marquina-Castillo et al.^12^ conducted in a mouse model. In their study, the Ph1 strain grew rapidly in the lung and peaked at 21 dpi, exhibiting a bacterial burden (via CFU readings) twice as high in comparison to those mice infected with the H37Rv strain.^12^ We infer this phenotype is likely due to the capacity of the Ph1 strain to colonise and grow within other leukocytes besides DCs, because, compared to H37Rv, the Ph1 strain displays similar *in vitro* phenotypes in terms of cord formation, growth curves, and response to hydrogen peroxide exposure.^12^ However, the authors did not examine the type of murine leukocytes serving as reservoirs for the bacteria. As previously reported, DCs exhibit a non-permissible phenotype against Mtb intracellular growth,^6^ and this characteristic is not influenced by the degree of virulence as we are showing in this study. Therefore, our results suggest that the Ph1 strain may have a better capacity to colonise macrophages in contrast to DCs.

Nevertheless, our observation that Ph1-infected DCs do not optimally activate autologous T cells is in line with the main finding in the study conducted by Marquina-Castillo et al.,^12^ which concluded that the Ph1 strain does not induce a protective immune response in a mouse model, such as the one promoted by the H37Rv strain. Indeed, infection of mice with the Ph1 strain was characterised by a delayed IFNγ expression, which is indicative of poor activation of Th1 cells.^12^ Infection of mice with other hypervirulent Mtb strains, such as those belonging to the W-Beijing lineage, also results in a poor protective Th1-driven immune response distinguished by the low and temporal expression of IFNγ, TNFα, and iNOS.^15^ ^,^ ^16^ Suboptimal antigen presentation has been shown to contribute to the virulence of Mtb in an *in vivo* mouse model.^17^ Moreover, DCs generated from monocytes after Type I IFN exposure (IFN-DCs) highly resemble naturally occurring DCs induced *in vivo*, for example, in a chronic infection context.^18^ In active TB patients, IFN-DCs showed a diminished capacity to induce Ag-specific T-cell responses against Mtb.^19^ Collectively, our results argue that the interaction of the Ph1 hypervirulent strain with human DCs differs from that of H37Rv, resulting in a lower capacity for these antigen-presenting cells to optimally activate an adaptive immune response.

The second key contribution of our study is the demonstration that the Ph4 hypovirulent strain induces apoptosis in human MDDCs, preventing them from supporting an autologous co-culture system with T lymphocytes. Similar to the cells infected with H37Rv, MDDCs were able to recognise, internalise, and control the intracellular growth of the Ph4 strain. However, MDDCs infected with this hypovirulent strain underwent rapid and massive levels of apoptosis, as compared to the other Mtb strains. These results suggest that less virulent Mtb strains can induce apoptosis in DCs, as well as macrophages, and in this manner, become a source of antigen for other antigen-presenting cells to pick up and promote an efficient adaptive immune response, as postulated previously.^7^ ^,^ ^20^ Interestingly, while the Ph4 strain did not differ in its capacity to activate MDDCs in terms of the up-regulation of cell surface receptors involved in antigen presentation (albeit always displaying the highest tendency among the tested strains), MDDCs infected with this strain were not able to support the established autologous co-culture system with CD4^+^ T cells. In the context of TB, infected migratory DCs are poor antigen-presenting cells,^21^ which suggests that infected cells have a mechanism to transfer antigen to uninfected cells so that uninfected cells could prime CD4^+^ T cells. *In vivo* studies demonstrated that migratory DCs must collaborate with one or more resident DCs to successfully prime CD4^+^ T cells.^22^ ^,^ ^23^ Cultured DCs and macrophages release multiple Mtb protein antigens into the extracellular medium that can be taken up, processed, and presented to CD4^+^ T cells by uninfected DCs.^23^ This form of antigen transfer occurs without transfer of the pathogen itself, providing a mechanism for host cells to bypass the inhibitory effects of Mtb on antigen presentation and allow for effective priming of antigen-specific CD4^+^ T cells. We inferred that MDDCs infected with the Ph4 strain become the source of mycobacterial antigens for uninfected bystander antigen-presenting cells, which may consequently activate an efficient T-cell response. Moreover, we believe that these results are in line with the characterisation performed in the mouse model by Marquina-Castillo and colleagues.^12^ Indeed, the authors determined that infection of mice with the Ph4 strain culminated in an increased survival rate of the mice in comparison to infection with the H37Rv strain. This was also correlated with a better protective acquired immune response characterised by high and sustained (albeit delayed) IFNγ expression, and early high levels of TNFα expression.^12^

It remains to be determined what signalling pathways promote the rapid and high levels of apoptosis triggered by the Ph4 strain in MDDCs. In the context of murine: bone marrow-derived-dendritic cells (BMDC) for example it was demonstrated that Mtb induces caspase-1/11-independent apoptosis but not necrosis.^7^ In the context of macrophages, avirulent Mtb strains induce strong expression of prostaglandin-endoperoxide synthase 2 (PTGS2), which consequently leads to high rates of apoptosis-promoting prostaglandin E2 (PGE_2_) production.^20^ Lipoxin A4 (LXA4) is known to inhibit PTGS2 and PGE_2_, leading to an increase in necrosis instead of apoptosis, which is common in macrophages infected by virulent Mtb strains.^5^ Altogether, our results argue that the interaction of the Ph4 hypovirulent strain with human DCs greatly differs from that of the H37Rv strain, resulting in rapid and massive rates of apoptosis that may facilitate the acquisition of antigenic material, thus leading hypothetically towards an efficient activation of the adaptive immune response.

In conclusion, this study represents the first prospective assessment of the human leukocyte response to the Mtb strains endemic in Southern Mexico that vary in virulence and transmission as previously assessed in mouse models. In general, our findings in human DCs suggest that the variability in virulence among these Mtb strains affects the capacity of leukocytes to respond to pathogenic challenge and mount an immune response against it, highlighting important parallels from studies performed in mouse models. The association of the observed effects in this study and specific virulence factors previously described for Mtb warrant further investigation. We support the notion that our Mtb clinical isolate strains make good candidates for further investigation using genome sequencing, transcriptome hybridisation, and comparative proteomics, probably leading to the eventual identification of Mtb genes associated with virulence and the interference of human DC biological functions, as previously proposed.^13^
